# Hypoxia-inducible factor 1 alpha expression increases during colorectal carcinogenesis and tumor progression

**DOI:** 10.1186/1471-2407-8-320

**Published:** 2008-11-04

**Authors:** Nektaria Simiantonaki, Marios Taxeidis, Caren Jayasinghe, Ursula Kurzik-Dumke, Charles James Kirkpatrick

**Affiliations:** 1 Institute of Pathology, Johannes Gutenberg University Mainz, Germany; 2 Institute of Medical Microbiology and Hygiene, Comparative Tumor Biology Group, Johannes Gutenberg University Mainz, Germany; 3 Institute of Pathology, Klinikum Leverkusen, Germany

## Abstract

**Background:**

Hypoxia-inducible factor 1 alpha (HIF-1α) is involved in processes promoting carcinogenesis of many tumors. However, its role in the development of colorectal cancer is unknown. To investigate the significance of HIF-1α during colorectal carcinogenesis and progression we examined its expression in precursor lesions constituting the conventional and serrated pathways, as well as in non-metastatic and metastatic adenocarcinomas.

**Methods:**

Immunohistochemistry and Western blot is used to analyse HIF-1α expression in normal colonic mucosa, hyperplastic polyps (HPP), sessile serrated adenomas (SSA), low-grade (TA-LGD) and high-grade (TA-HGD) traditional adenomas as well as in non-metastatic and metastatic colorectal adenocarcinomas. Eight colorectal carcinoma cell lines are tested for their HIF-1α inducibility after lipopolysaccharide (LPS) stimulation using western blot and immunocytochemistry.

**Results:**

In normal mucosa, HPP and TA-LGD HIF-1α was not expressed. In contast, perinuclear protein accumulation and nuclear expression of HIF-1α were shown in half of the examined SSA and TA-HGD. In all investigated colorectal carcinomas a significant nuclear HIF-1α overexpression compared to the premalignant lesions was observed but a significant correlation with the metastatic status was not found. Nuclear HIF-1α expression was strongly accumulated in perinecrotic regions. In these cases HIF-1α activation was seen in viable cohesive tumor epithelia surrounding necrosis and in dissociated tumor cells, which subsequently die. Enhanced distribution of HIF-1α was also seen in periiflammatory regions. In additional *in vitro *studies, treatment of diverse colorectal carcinoma cell lines with the potent pro-inflammatory factor lipopolysaccharide (LPS) led to HIF-1α expression and nuclear translocation.

**Conclusion:**

We conclude that HIF-1α expression occurs in early stages of colorectal carcinogenesis and achieves a maximum in the invasive stage independent of the metastatic status. Perinecrotic activation of HIF-1α in invasive tumors underlines a dual role of HIF-1α by regulating both pro-survival and pro-death processes. HIF-1α up-regulation in response to LPS-mediated stimulation and periinflammatory expression in invasive carcinomas suggest its involvement in inflammatory events. These patterns of HIF-1α inducibility could contribute indirectly to the acquisition of a metastatic phenotype.

## Background

As a consequence of increased cellularity and proliferation, as well as enhanced metabolism within a tumor, the oxygen concentration in solid neoplasms is generally lower than in the adjacent non-neoplastic tissue [1,2]. In fact, histopathological examination of carcinomas frequently reveals hypoxic areas within the tumor mass, mostly in the form of necrotic regions. As a reaction to hypoxia tumor cells can alter their metabolism and activate survival mechanisms. Hypoxia inducible factor 1 (HIF-1) is a transcription complex that plays a crucial role in coordinating the cellular response to oxygen stress conditions [3]. As a result of hypoxic stress, HIF-1 activates the transcription of a variety of genes regulating cell survival. HIF-1 is a heterodimer composed of one of the three alpha subunits (HIF-1α, HIF-2α or HIF-3α) and one HIF-1β subunit. Although HIF-1β is constitutively expressed, hypoxia-mediated responses are determined by HIF-α subunits. In normoxia, degradation of HIF-1α occurs. Hypoxia leads to HIF-1α stabilization, resulting in a translocation of HIF-1α to the nucleus and binding to HIF-1β forming the active HIF-1 complex. Activation of HIF-1α has been reported in many solid tumors including carcinomas of the gastrointestinal tract [4-12]. However, the role of HIF-1α in tumor progression of colorectal carcinomas is still unclear and the published data so far are controversial. Thus, the expression of HIF-1α in some studies was correlated with an increased tumor aggressiveness, whereas in other studies a direct contribution to tumor progression was not found.

HIF-1α is one of the key factors promoting carcinogenesis independently of histogenetical origin. An enhanced expression of HIF-1α from normal tissue through premalignant lesions to carcinomas has already been observed in prostate [13], gastric [14], breast [15], oral cavity [16], cervical [17] and endometrial [18] carcinogenesis. So far, detailed studies about the role of HIF-1α in the development of colorectal cancer do not exist. In the case of colorectal carcinogenesis two major pathways with different morphological features of the precursor lesions has been described [19,20]. One is the convensional "adenoma-carcinoma pathway" and the other is the alternative "serrated pathway", in which serrated polyps replace the traditional adenoma as the precursor lesion. The incidence of colorectal cancer did not differ significantly between serrated and traditional adenoma.

In addition to hypoxia, more recent evidence suggests that non-hypoxic pro-inflammatory stimuli, including cytokines and growth factors, can also activate HIF-1α under normoxic conditions and modulate the transcription of hypoxia-associated genes [21]. This phenomenon is well documented in inflammatory cells such as macrophages and monocytes [22-24] but similar observations have also been made in tumor cell lines [25,26]. In this context, the very potent pro-inflammatory bacterial lipopolysaccharide (LPS) may be of particular importance as a possible stimulator of HIF-1α in the gut under normoxic conditions. Because LPS is a component of the cell wall of gram-negative bacteria ubiquitously present in the colon, normal and neoplastic intestinal cells are continuously exposed to this factor [27]. Our previous studies suggest that LPS could influence the progression of colorectal cancer through its receptor TLR4 (Toll-like receptor 4) [28] and the downstream transcription factor NFκB (nuclear factor-κB) [29], leading to release of factors from colon carcinoma cells capable of up-regulating endothelial cell adhesion molecules [30]. Interestingly, a study investigating gut ischemia, has found that LPS can induce HIF-1α expression in enterocytes under normoxic conditions [31]. However, a potential activation of HIF-1α in colorectal carcinoma cells after exposure to LPS has not been reported.

On the basis of the above considerations, in the present study we set out to investigate the expression and role of HIF-1α in colorectal cancer development and progression. To determine the potential role of HIF-1α in colorectal carcinogenesis we evaluated its expression in classical and serrated premalignant lesions and in normal mucosa. To examine whether HIF-1α can influence the progressive behavior of colorectal carcinomas, we compared its expression in non-metastatic and metastatic malignant cases. Additionally, we verified its stimulation in colorectal carcinomas under normoxic inflammatory conditions by determining its inducibility after LPS stimulation in various colorectal carcinoma cell lines.

## Methods

### Cell culture conditions and LPS stimulation experiments

The human colorectal carcinoma cell lines SW837, HRT18, CX-1, CX-2, SW620, SW948, HT-29 and CaCo2 were grown in RPMI 1640 medium supplemented with Glutamax (Sigma, St. Louis, MO, USA), 10% heat-deactivated FCS (Gibco BRL, UK), 1% penicillin (Gibco) and 1% streptomycin (Gibco) at 37°C with 5% CO_2_. LPS stimulation was performed by replacing the growth medium of subconfluent cultures with medium supplemented with LPS derived from E. coli (Sigma), 1 μg/ml, for 4 hours. After stimulation cells were harvested and used for expression analysis.

### Tissue samples

Colorectal polyp tissue were obtained from patients undergoing colonoscopy with biopsy extraction at the Klinikum Leverkusen during 2005–2007. In 20 of the biopsy samples normal mucosa was also available for observation. Colorectal carcinoma tissue samples were obtained from 92 patients undergoing elective surgery at the University of Mainz during the years 1995–1999. The investigation of these tissues was in accordance with the rules of the responsible state ethical committee of the Mainz University. All investigated tumors were T3 (subserosal infiltration) and moderately differentiated (G2) adenocarcinomas. This selection was performed with regard to a potential relationship between the expression level of HIF-1α and the metastatic status of the tumors. With regard to the metastatic stage 38 cases were without tumor metastasis to regional lymph nodes or distant organs (N0/M0) during a follow-up period of at least 5 years after surgery, 33 of the cases were characterized by haematogenous (M+) and 21 by lymphogenous metastases (N+).

### Western blotting

Protein extracts from harvested culture cells as well as normal and tumor epithelia derived from patients undergoing surgical resection of colorectal cancer were used. From each sample aliquots containing 20 μg of total protein were separated on sodium dodecyl sulphate-polyacrylamide gels SDS-PA (10%) and then transferred to polyvinylfluoride (PVDF) membranes (Immobilen-P, Millipore Corp.) in accordance with standard procedures. Incubation with the primary antibodies to mouse monoclonal anti-HIF-1α (1:100) (Stressgen, Victoria, BC Canada), and rabbit polyclonal anti-β-actin (1:100) (Serva, Heidelberg, Germany) was performed overnight by 4°C. Immunodetection was performed using the alkaline phosphatase (AP) conjugated anti-mouse and anti-rabbit IgG (1:1000) (Sigma).

### Immunohistochemistry/cytochemistry

All immunohistochemical reactions were conducted using formalin-fixed and paraffin-embedded samples. After deparaffinization the samples were treated in a microwave oven in EDTA buffer for 15 minutes. For immunocytochemistry cytospins were prepared. Subsequently, fixation in an mixture of acetone and 4% formalin was carried out. Incubation with the primary antibody to HIF-1α (1:100) (Stressgen) and the secondary antibody, horse anti-mouse biotinylated IgG (1:200) (Vector, Burlingame, CA, USA) was carried out in accordance with standard protocols using the Vectastain Elite reagent (Vector). Sections were counterstained with Mayer's hematoxylin. To prove the specificity of the immunoreactions every sample was stained solely with the secondary antibody. This immunoreaction was in every case completely negative. The additionally required check of antibody specifity by HIF-1α lacking cells is demonstrated in the non-hypoxic, and non-inflammatory colonic mucosa where none of the cells express HIF-1α.

### Evaluation of HIF-1α staining

Immunostaining reactions of each sample were evaluated by three authors independently (N.S., C.J., M.T.). Specific immunoreactivity was observed in the cytoplasm and in nuclei of the tumor cells. Since cytoplasmatic staining was observed homogeneously in the tumor cells, the cases were classified according to staining intensity in weakly, moderately, and strongly positive. Nuclear immunoreactivity was heterogeneous in the tumor, so that the score was ascertained by consideration of both staining density and intensity. The density of nuclear staining was classified as follows: 1, nuclear staining in less than 20% of cells; 2, nuclear staining in 20–40% of cells; 3, nuclear staining in more than 40% of cells. Nuclear staining intensity was estimated as 1, weak and 2, strong. The multiplication of these two values gave the final score for nuclear staining: 1–2, weak; 3–4, moderate; 5–6, strong.

### Statistical analysis

The association of staining intensity with tumor stage was assessed with χ^2 ^(Fisher's exact test). P < 0.05 was considered to be significant in all statistical analyses.

## Results

### HIF-1α expression in non-neoplastic and premalignant colorectal lesions

**Figure 1:**
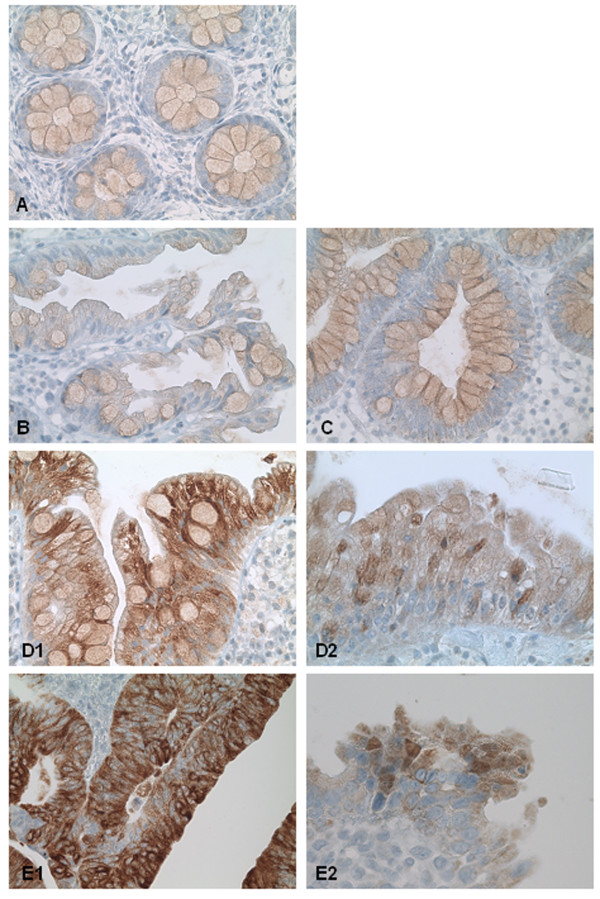
**HIF-1α expression in precursor lesions of colorectal carcinogenesis. **In normal mucosa (A), HP (B) and TA-LGD (C) specific HIF-1α expression was not seen. SSA and TA-HGD showed a strong diffusely perinuclear (D1 and E1) and on the surface crypts focally nuclear (D2 and E2) HIF-1α immunopositivity.

In the case of colorectal carcinogenesis two different pathways with distinct morphological features of the precursor lesions has been described [18,19]. In the classical pathway the precursor lesion is the traditional adenoma (TA), with variable degrees of dysplasia, ranging from low grade (TA-LGD) to high grade (TA-HGD). In the serrated pathway the histopathological sequence begins with the hyperplastic polyp (HP) without malignant potential, followed by the sessile serrated adenoma (SSA), recognised as a precursor lesion. A summary of the HIF-1α immunostaining profile in non-neoplastic tissue and in precursor lesions of colorectal carcinogenesis is provided in Table 1. Normal colonic mucosa, HP and TA-LGD showed neither specific cytoplasmic nor nuclear HIF-1α expression (Figure 1A–C). In these samples the mucus content of the colonic goblet cells leads to a non-specific immunoreactivity. In contrast, SSA (7 of 13 samples) showed a strong, diffusely perinuclear HIF-1α expression with nuclear immunopositivity in about 10% of the cells (Figure 1D1,2 ). Additionally, TA-HGD (10 of 20 samples) showed a strong perinuclear, cytoplasmatic and focally nuclear positive immunostaining for HIF-1α. (Figure 1E1,2). In both lesions, the intense immunoreactivity was observed on the surface but not in the lower crypts. **I**

**Table 1:** HIF-1α expression in benign and premalignant colorectal lesions

	**n**	**HIF-1 positivity**
normal mucosa	20	0
HP	20	0
TA-LGD	20	0
TA-HGD	20	10 (50%)
SSA	13	7 (54%)

Abbreviations: HP – hyperplastic polyp, TA-LGD – tradtional adenoma-low grade dysplasia, TA-HGD – tradtional adenoma-high grade dysplasia, SSA – sessile serrated adenoma

### HIF-1α expression in non-metastatic and metastatic colorectal carcinomas

**Figure 2:**
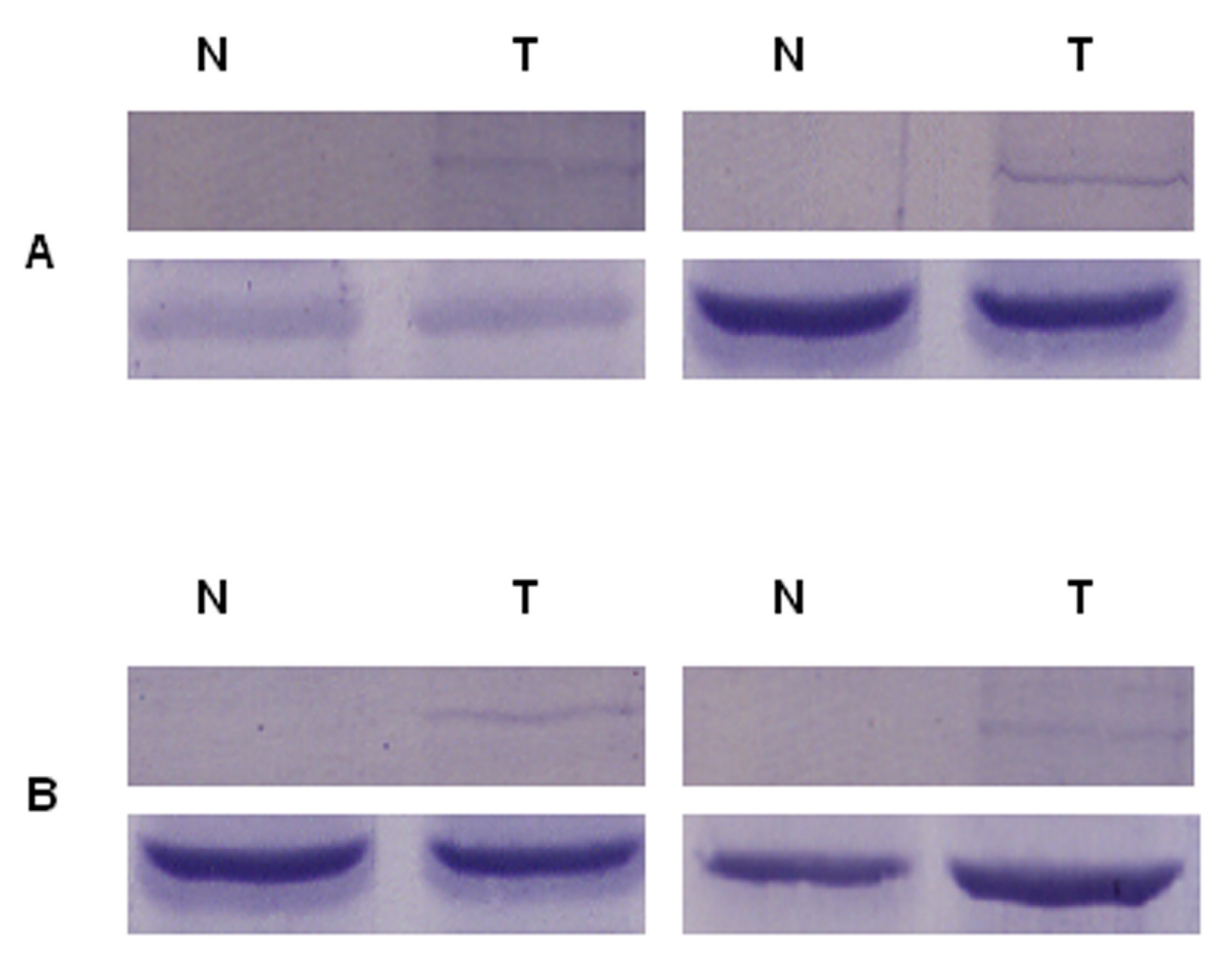
**Detection of HIF-1α in human non-metastatic (A) and metastatic (B) primary colorectal carcinomas by Western blot.** Staining with anti-β-actin (bottom blots respectively) was performed as control for loading. (N: corresponding normal epithelium; T: tumor epithelium).

HIF-1α protein expression in neoplastic tissue, but not in the adjacent normal mucosa was confirmed by western blot analysis of each of two non-metastatic and metastatic colorectal carcinomas (Figure 2).

**Figure 3:**
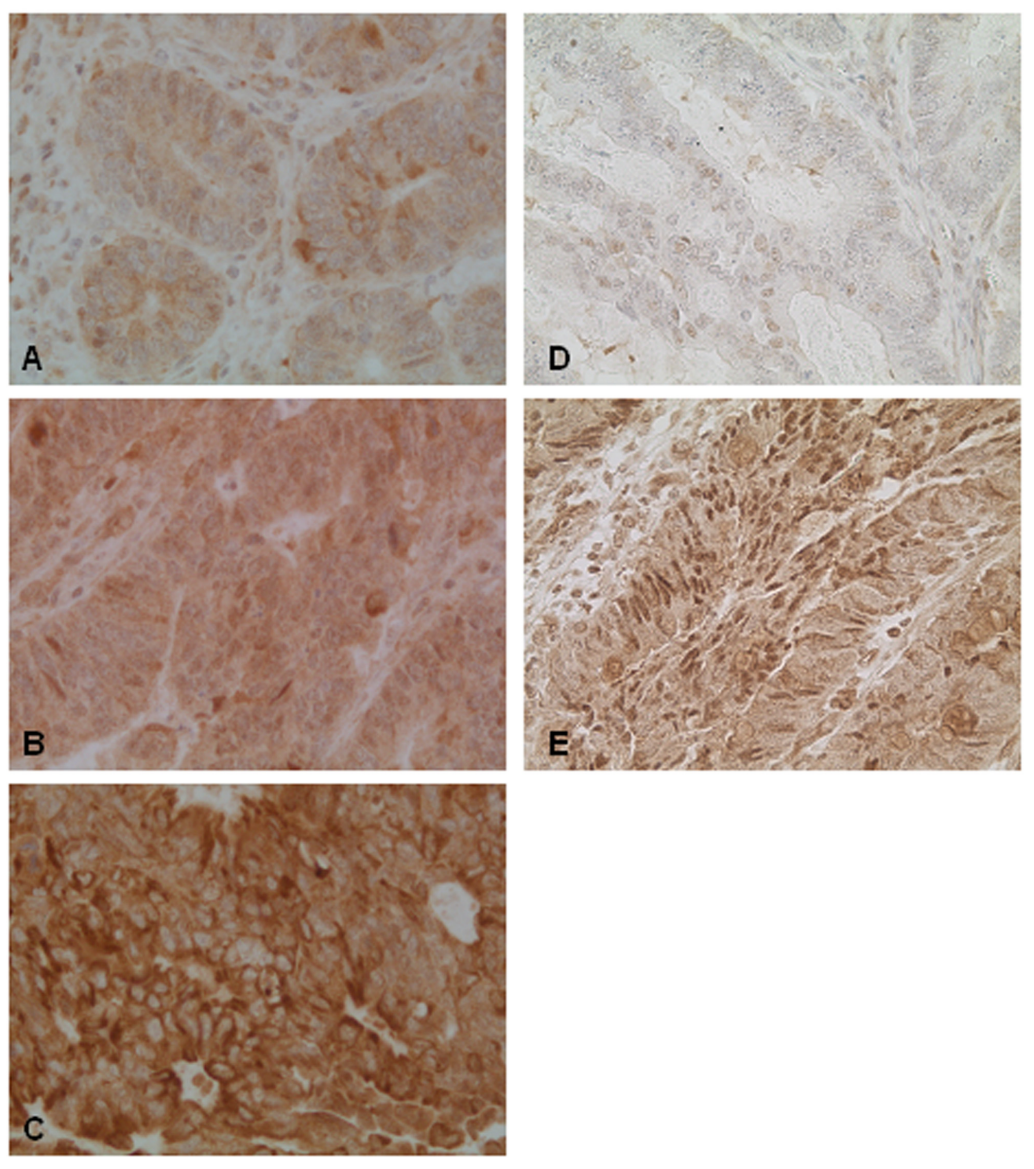
**Cytosolic (A-C) and nuclear (D and E) HIF-1α expression profiles detected in colorectal carcinomas.** With respect to the cytoplasmic expression intensity weak (A), moderate (B) and strong (C) immunoreactivity was detected. With respect to the nuclear expression intensity weak (D) and strong (E) immunoreactivity was seen.

**Figure 4:**
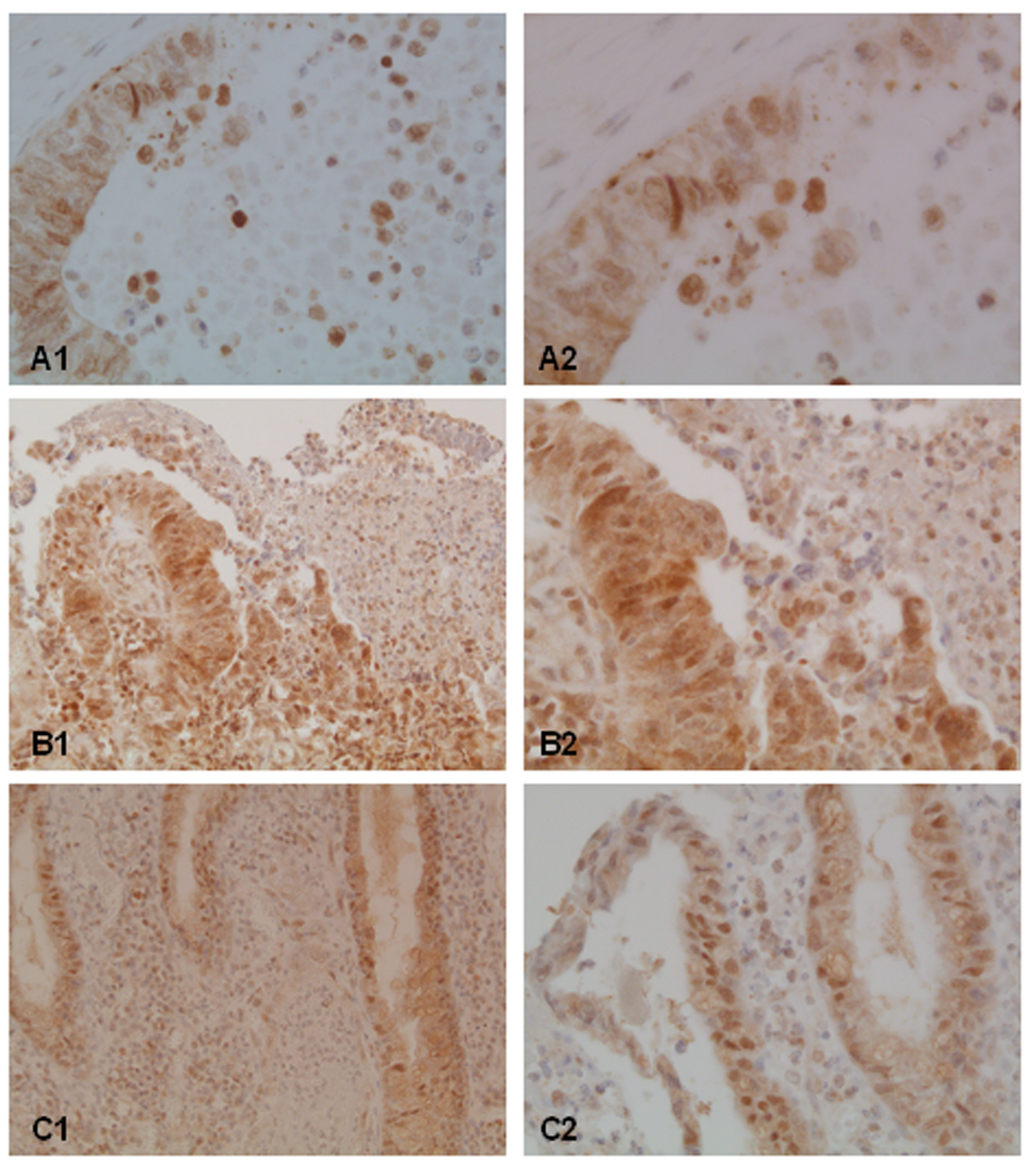
**Accumulation of nuclear HIF-1α in perinecrotic (A1,2) and periinflammatory (B1,2 and C1,2) regions of the tumor tissue. **In perinecrotic areas HIF-1α activation was seen in cohesive tumor epithelia surrounding necrosis and in dissociated, dying tumor cells. In periiflammatory areas tumoral HIF-1α activation was seen in the superficial tumor ulceration (B1,2) and in severe inflammation along the infiltrative pathway (C1,2). Magnification: 1 = 200×, 2 = 400×

Immunohistochemical expression of HIF-1α was observed in the cytoplasm and in the nucleus of the tumor cells in all investigated cases but with different staining intensity (Figure 3). As shown in table 2 there was no significant correlation in the cytoplasmatic or nuclear expression between non-metastatic tumors and carcinomas with lymph node and distant metastases. Topological differences of immunoreactivity between the superficial tumor fraction and the invasive tumor edge were not seen. The cytosolic staining distribution in each specimen was widely homogenous. In contrast, the distribution of the number of positive tumor nuclei and the nuclear immunostaining intensity of HIF-1α was heterogeneous but without significant differences according to the metastatic status (Table 2). However, the distribution of the active, endonuclear HIF-1α form in the tumor was not haphazard, but strongly associated with histological features such as necrosis and severe peritumoral inflammation. 65 of the 92 tumors examined were characterized by abundant central necrosis in the atypical glands accompanied by inceased HIF-1α immunoreactivity both in density and intensity in the vicinity of these areas in 55 cases (85%) (Figure 4A). In these samples nuclear HIF-1α expression was detected in cohesive tumor epithelia surrounding necrosis and in dissociated cells destined to undergo cell death (Figure 4A1,2). 38 cases of the examined 92 tumors showed a severe peritumoral inflammation, characterized by dense confluent, mixed and mainly granulocyte-rich inflammatory infiltrates. The inflammation was located in the superficial tumor ulceration and along the infiltrative pathway of the tumor. In 28 of these cases (74%) an intensive nuclear HIF-1α expression of surrounding tumor cells was observed (Figure 4B1,2 and 4C1,2).

**Table 2:** HIF-1α expression in non-metastatic (N0/M0), lymphogenously-metastatic (N+) and haematogenously-metastatic (M+) colorectal carcinomas

		**cytoplasmatic HIF-1α expression**			**nuclear HIF-1α expression**		
			
**metastatic status**	**n**	**weak**	**mod**	**strong**	**weak**	**mod**	**strong**
N0/M0	38	5	12	21	8	6	24
N+	21	5	6	10	3	10	10
M+	33	6	11	16	3	17	13

With respect to the T status all investigated tumors were T3 and moderately differentiated (G2). No statistical significance between cytoplasmtic or nuclear HIF-1α expression and the metastatic status was observed.

### Non-hypoxic HIF-1α stimulation by the pro-inflammatory signal, LPS, in colorectal carcinoma cell lines

**Figure 5:**
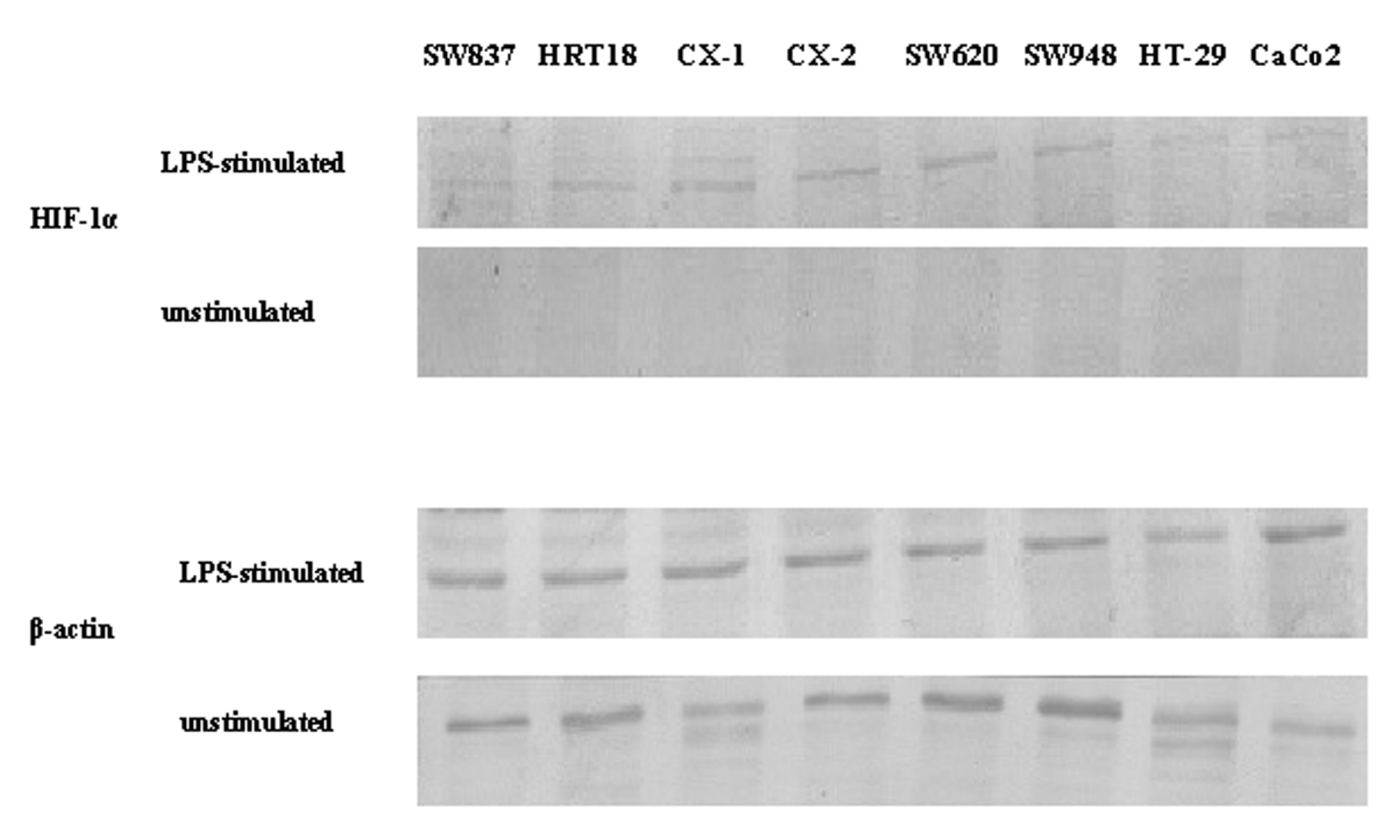
**Detection of HIF-1α in unstimulated and LPS-stimulated human colorectal carcinoma cell lines by Western blot.** Staining with anti-β-actin was performed as control for loading.

**Figure 6:**
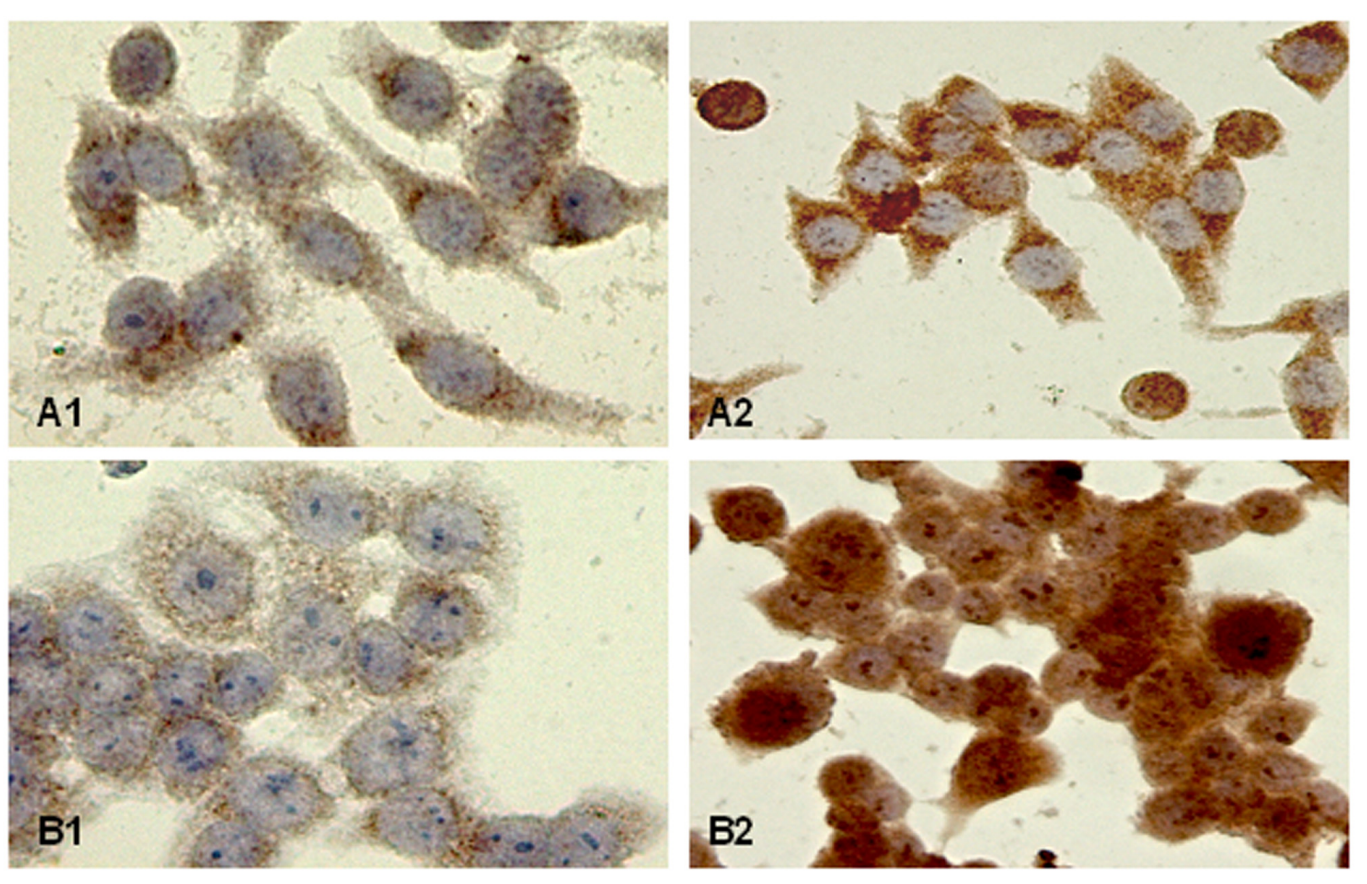
Positive cytoplasmic and nuclear expression of HIF-1α in LPS-stimulated SW620 (A2) and HRT-18 (B2) colon carcinoma cell lines compared to native conditions (A1 and B1).

Since a strong association between nuclear expression of HIF-1α and severe peritumoral inflammation was observed *in situ*, the question arises whether LPS stimulation of colorectal carcinoma cell lines leads to up-regulation of this transcriptional factor. In this context we examined by Western blot HIF-1α expression in diverse unstimulated and LPS-stimulated colorectal cell lines – SW837, HRT18, CX-1, CX-2, SW620, SW948, HT-29 and CaCo2. The LPS-stimulation experiments were conducted under normoxia (21% O_2_). As shown in Figure 5 all eight cell lines were negative for HIF-1α in the native condition. Interestingly, LPS stimulation of these cell lines resulted in HIF-1α up-regulation. Since the active form of HIF-1α is located in the nucleus we next examined its subcellular localization by immunocytochemistry (Figure 6). LPS-treatment of the colorectal carcinoma cell lines was due to enhanced perinuclear accumulation and nuclear translocation of HIF-1α 4 hours after stimulation.

## Discussion

The present study represents a systematic morphological investigation of HIF-1α in colorectal cancer development. So far, only one study reported detection of HIF-1α m-RNA in four of nine investigated adenomas [7]. Our analysis demonstrated a significant induction of HIF-1α in half of the cases of the premalignant lesions SSA and TA-HGD. In contast, in normal mucosa, in benign HP and in TA-LGD, which is known to have only a low risk of malignant transformation HIF-1α expression was absent. Since the investigated preneoplastic lesions did not show any necrosis that expression is independent of hypoxia. Due to the fact that in benign tissue HIF-1α is not detected, the expression in tumor tissue could arise from genetic alterations. In fact, HIF-1α is involved in tumor progression by promoting genomic instability [32]. In the most common traditional pathway of colorectal carcinogenesis the chromosomal instability by stepwise mutation of *Ki-ras*, *APC*, and *p53 *genes generally leads to development of approximately 70%–85% of colorectal carcinomas [33]. Whether an interactive correlation exists between *Ki-ras *or *APC *and HIF-1α activity status still remains to be determined. However, induction of HIF-1 activity by loss-of-function mutations of the tumor suppressor gene *p53 *has already been observed [34]. Sugai et al reported about progressive increase in mutations of the *p53 *gene in colorectal adenomas from low-grade to high-grade [35]. In this context, we suggest that this observation could be associated with our observations of HIF-1α upregulation in the same stage of colorectal carcinogenesis. The serrated pathway shown in about 15% of the colorectal carcinomas demonstrates *BRAF *mutation and methylation/silence of the *MLH-1 *mismatch repair complex in the context of microsatellite instability [34]. Interestingly, *BRAF *mutations are rare in HP (2%) but very frequent in SSA (75%) [36-38]. Notably, a recent study with melanoma cells has shown for the first time that *BRAF *mutation increases HIF-1α expression [39]. *MLH-1 *methylation has been detected in 36% of HP and in 70% of SSA [40,41]. *MLH-1 *gene was downregulated in hypoxia but a direct involvement of HIF-1α has not been found [42]. We conclude that overexpression of HIF-1α can occur early in colorectal carcinogenesis in both the traditional (adenoma-carcinoma seqence) and serrated pathway. We suppose that this HIF-1α activation is associated with the stepwise progressive genetic instability during colorectal cancer development. Further investigations are necessary to elucidate the precise mechanisms of this interaction.

In a previous *in vitro *study with the human colon carcinoma cell line HCT-116 it was demonstrated that HIF-1α regulates the expression of genes encoding proteins involved in the pathophysiology of tumor invasion [43]. In our present *in situ *study, in comparison with the adenomas in colorectal adenocarcinomas a conspicuous increase in density and intensity of HIF-1α expression was found, reflecting a gradual increase in expression level throughout the sequence of intestinal carcinogenesis with a maximum in the stage of the invasive phenotype. The distinctly elevated HIF-1α expression in malignant tissue allows the assumption of a relevant contribution of the characteristic hypoxic microenvironment. In all investigated cases a positive HIF-1α expression was seen with cytosolic and nuclear localization, consistent with synthesis in the cytoplasm and subsequent translocation to the nuclei. Although the cytoplasmic staining distribution was homogenous, nuclear staining distribution was not uniform and showed an accumulated presence in the vicinity of tumor necrosis and the neighborhood of severe peritumoral inflammation. This topological pattern of the transcriptionally active form provides evidence that HIF-1α could play a crucial role in conditions characteristic of tumoral microenvironment, such as necrosis and inflammation, giving low levels of oxygen and high levels of inflammatory cytokines. Interestingly, in the perinecrotic regions nuclear positivity was seen in cohesive tumor epithelia of the atypical glands surrounding areas of necrosis but also in dissociated cells within necrosis. According to our view, these histopathological findings underline the dual, apparently paradoxical role of HIF-1α in regulating both pro-survival and pro-death processes [44]. HIF-1α activation in the cohesive tumor cells could be explained as transduction to an adequate adaptative intracellular response to prevent from cell death. Simultaneously, HIF-1α activation in the dissociated tumor cells could be explained as initiation of a "one way" cascade leading to cell death.

Our study adds new data to support the concept of HIF-1α involvement in normoxic inflammatory processes. HIF-1α activation in colorectal cancer specimens was seen in the vicinity of severe inflammation located in the superficial tumor ulceration and along the invasion pathway. Due to the fact that intestinal mucosal injury may permit translocation of bacteria and endotoxin, this topological accumulation of the active form of HIF-1α could be of great importance for tumor progression [45]. Strong direct evidence suggests that cancer-associated inflammation promotes tumor growth and progression [46]. In the past, we identified LPS, an endotoxin of ubiquitously existing colonic bacteria, as a pivotal pro-inflammatory stimulus increasing the aggressive behavior of colorectal carcinomas [28-30]. We report here that LPS stimulation of diverse colorectal carcinoma cell lines induced HIF-1α expression and translocation to the nucleus. These findings demonstrate that LPS is a non-hypoxic stimulus for HIF-1α in colorectal carcinomas. Based on our results we hypothesize that HIF-1α could function as a linking transcription factor for LPS-mediated processes along the tumor invasion from the luminal site to the site of deepest penetration.

In an *in vitro *study we recently reported that HIF-1α upregulation in response to hypoxia was accompanied by a significantly increased production of vascular endothelial growth factor (VEGF) in two of five colorectal carcinoma cell lines [47]. Kuwai et al reported an hypoxia-induced increase of HIF-1α and VEGF in three of the four examined colorectal carcinoma cell lines [6]. These researchers and others described a strong association between HIF-1α and VEGF expression in colorectal carcinoma specimens [6,7,10]. Since VEGF promotes lymphangiogenesis and angiogenesis in the primary tumor, thereby paving the way for metastasis, these findings suggest that HIF-1α plays a determinative direct role in the processs of colorectal cancer metastasis. However, in our current *in situ *investigations there were no significant differences in the HIF-1α expression levels between non-metastatic tumors and carcinomas with lymph node and distant metastasis. The published data so far concerning a correlation between HIF-1α expression and metastatic disease status of colorectal carcinomas are controversial. In accordance with our results Joshimura et al [8] and Wincewicz et al [12] found that HIF-1α expression did not differ in relation to metastatic stage. In contrast, in two other studies a significantly higher expression of HIF-1α was observed in Dukes stages C or D, characterized by a positive status for nodal or distant metastases [7,11]. Lu et al [10] showed that HIF-1α expression was strongly associated with lymph node metastasis, whereas Kuwai et al [6] report a significant correlation with liver but not with lymph node metastasis. Because of the chosen design in this study with comparison of non-metastatic and metastatic colorectal carcinomas with the same grading of differentiation and infiltrating depth, the influence of both essential tumor features on the HIF-1α expression can be excluded. In previous *in vitro *experiments we demonstrated the relevance of tumor differentiation for HIF-1α inducibility [47]. Well-differentiated colorectal carcinoma cell lines were hypoxia-resistent, showing unchanged levels of HIF-1α and VEGF in response to reduced oxygen stress conditions.

## Conclusion

**Figure 7:**
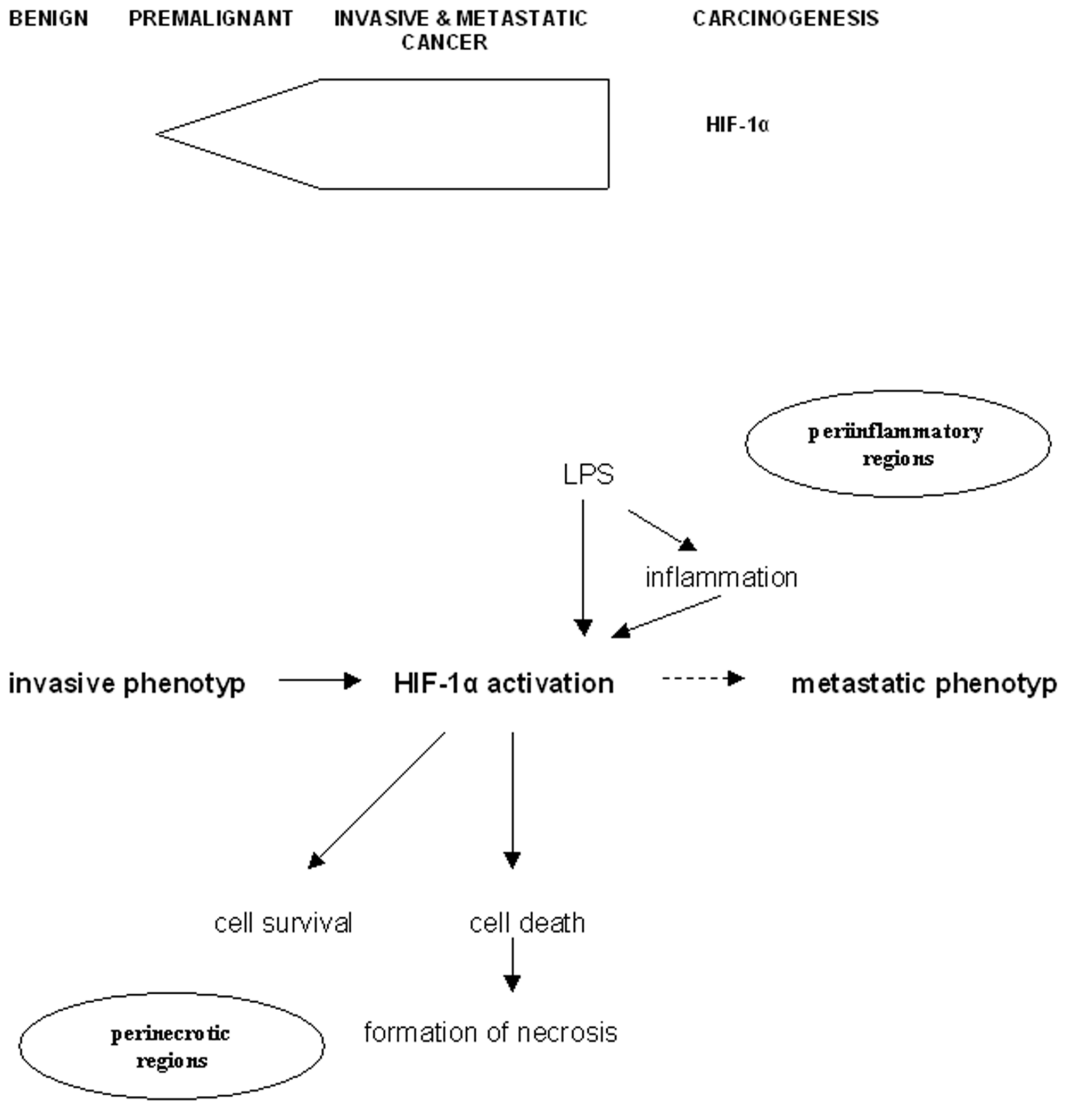
Schematic illustration of the HIF1α-mediated pathway in colorectal carcinogenesis and tumor progression.

We conclude that HIF-1α expression occurs in early stages in the classical and serrated pathway of colorectal carcinogenesis and achieves a maximum in the invasive stage, independent of the metastatic status of the primary tumor (Figure 7). Since the acquisition of metastatic phenotype is a multistep event, the HIF-1α associated perinecrotic processes of both tumor cell survival and death and LPS-mediated inflammatory response could indirectly contribute to this endpoint.

## Abbreviations

HIF-1: hypoxia inducible factor 1; HP: hyperplastic polyp; TA-LGD: tradtional adenoma-low grade dysplasia; TA-HGD: tradtional adenoma-high grade dysplasia; SSA: sessile serrated adenoma; N0/MO: non-metastatic colorectal adenocarcinomas; N+: lymphogeneously metastatic colorectal adenocarcinomas; M+: haematogeneously metastatic colorectal adenocarcinomas; LPS: bacterial lipopolysaccaharide; VEGF: vascular epithelial growth factor; APC: adenomatous polyposis coli; MLH-1: mutL homolog 1.

## Competing interests

The authors declare that they have no competing interests.

## Authors' contributions

NS carried out the design of the study and western blotting, participated in analysis of immunostaining, performed the statistical analysis and wrote the manuscript; MT carried out the immunohistochemistry and participated in analysis of immunostaining; CJ carried out the *in vitro *experiments and the immunocytochemistry and participated in analysis of immunostaining; UKD helped in western blotting; CJK helped in critical review of the manuscript. All authors read and approved the final manuscript.

## Pre-publication history

The pre-publication history for this paper can be accessed here:


